# Supplementary material for: Human CD4-binding site antibody elicited by polyvalent DNA prime-protein boost vaccine neutralizes cross-clade tier-2-HIV strains
Source: Nat Commun. 2024 May 21;15:4301. doi: 10.1038/s41467-024-48514-8 (PMC11109196; doi:10.1038/s41467-024-48514-8)
Supplement: Supplementary file 1 — Supplementary Information [file 41467_2024_48514_MOESM1_ESM.pdf]

a

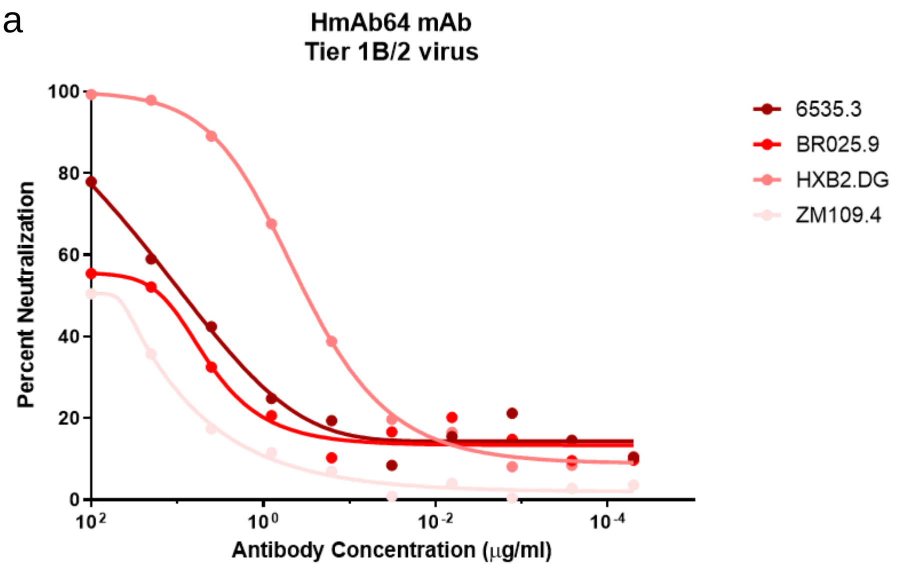

b

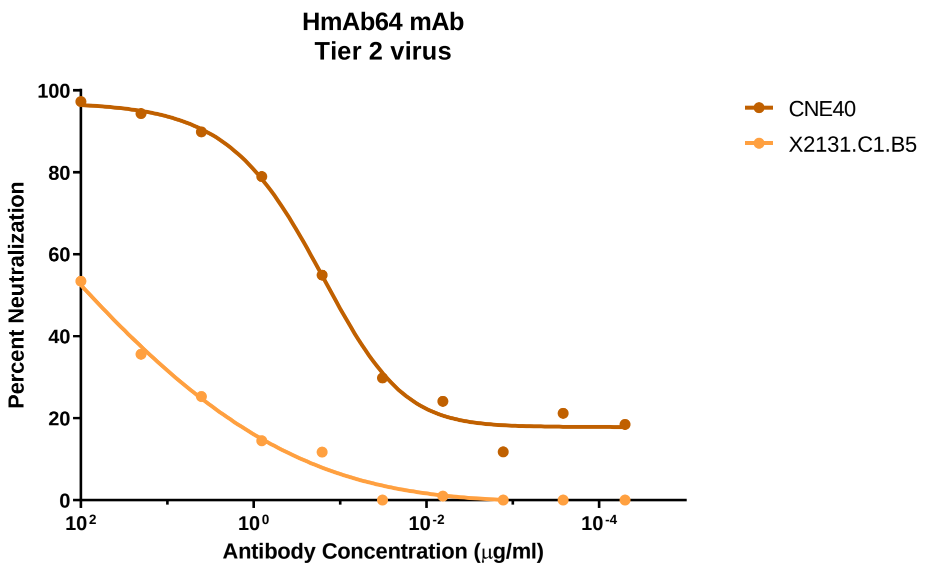

Supplementary Figure 1 | Neutralization curves for Tier 1B/2 and Tier 2 viruses neutralized by HmAb64.

(a) Four tier 1B/2 viruses from 208-strain NIAID VRC panel neutralized by HmAb64.

(b) Two tier 2 viruses from 208-strain NIAID VRC panel neutralized by HmAb64.

Source data are provided as a Source Data file.

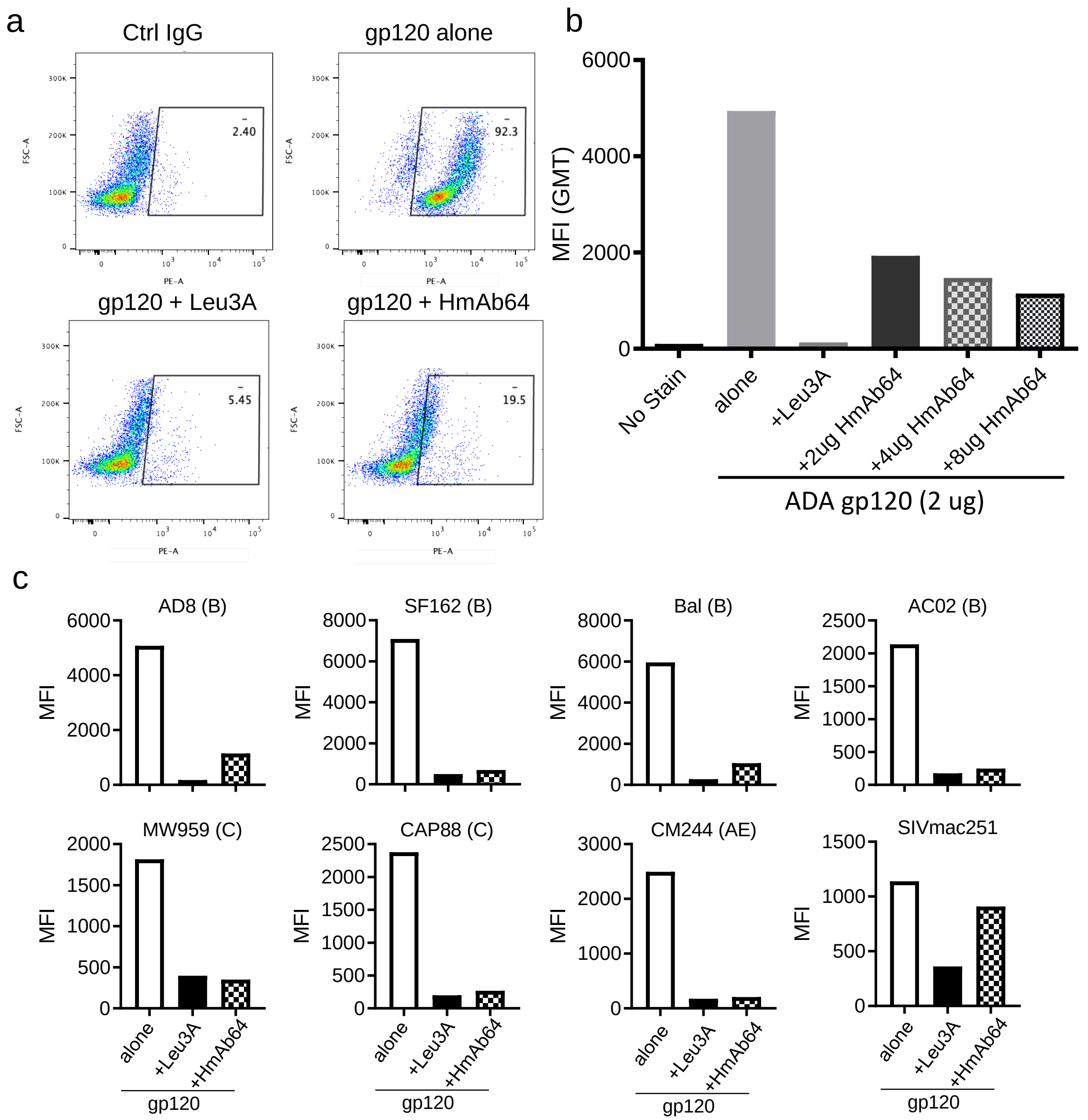

Supplementary Figure 2 | HmAb64 blocked binding of gp120 Env proteins to human CD4+ T cells in a FACS-based assay.
(a) HmAb64 blocked binding of gp120 to CD4+ T cells. CD4-binding site antibody Leu3A was used as a positive control.
(b) HmAb64 blocking is dose dependent.
(c) HmAb64 blocked binding of diverse gp120 antigens to CD4+ T cells. Env protein from SIVmac251 was used as a negative control.

Source data are provided as a Source Data file.

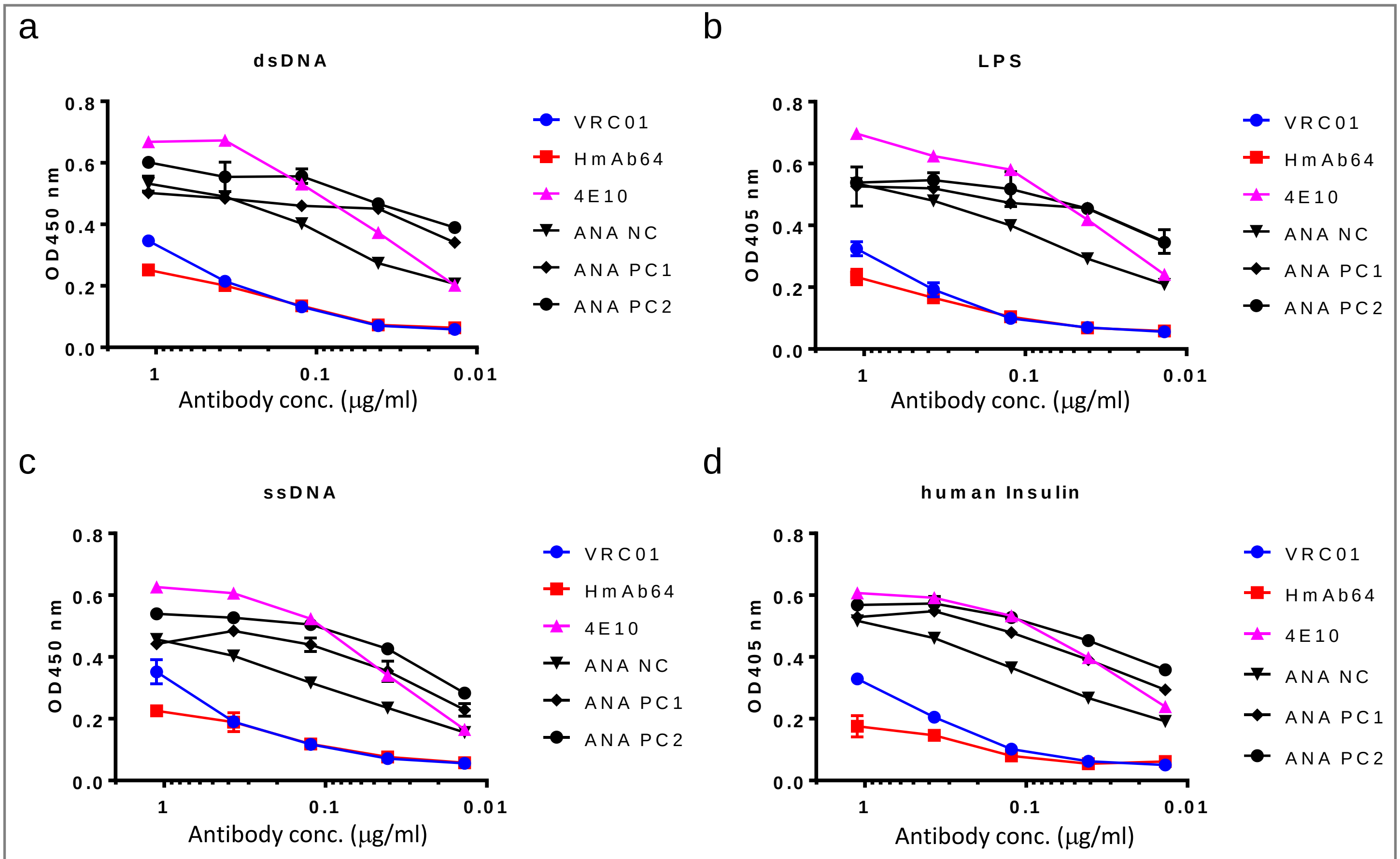

Supplementary Figure 3 | Test of HmAb64 binding to various auto-antigens.

The ability of HmAb64 and other control mAbs and reagents binding to various known auto-antigens were tested by ELISA including (a) dsDNA, (b) LPS, (c) ssDNA and (d) human insulin.

Source data are provided as a Source Data file.

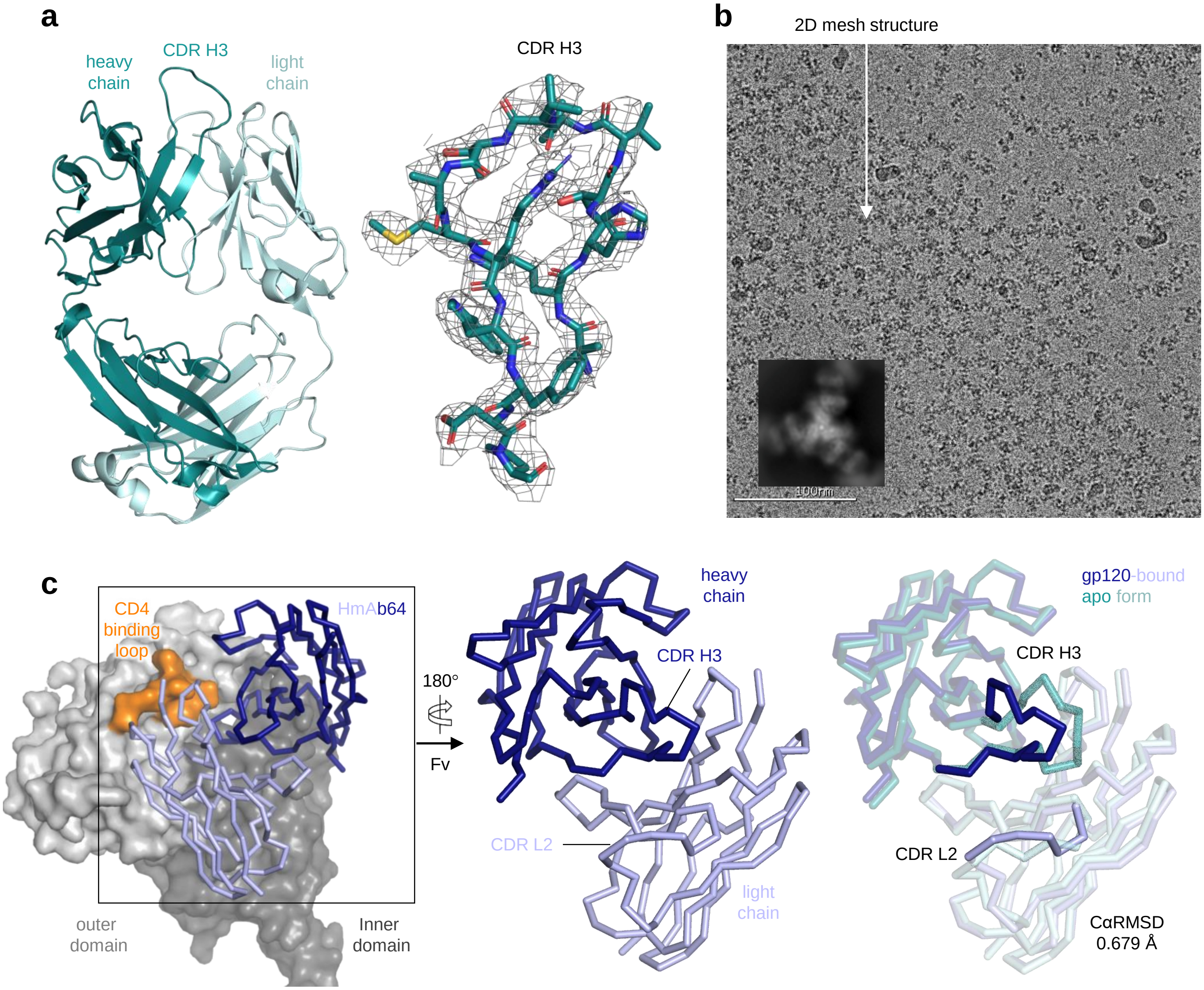

Supplementary Figure 4 | Structure of HmAb64 Fab and comparison between free and ligand-bound HmAb64. a Crystal structure of the apo form of HmAb64 Fab (in ribbons, PDB 6W73) and its CDR H3 loop (in sticks) with the 2Fo-Fc density map (in gray meshes) generated in PyMOL at the contour level of 0.6. **b** Representative cryo-EM micrograph of the HmAb64 Fab in complex with the SOSIP trimer revealed the formation of a 2D honeycomb-like lattices, likely caused by the interaction between Fabs, as depicted in the inset, which presents a 2D average image (6,596 selected particles) of the complex within the lattice structure. **c** Structural comparison between free and ligand-bound HmAb64 was performed by aligning the variable regions of the apo form HmAb64 Fab (in teal) and the gp120-bound scFv (in blue) in PyMOL, with RMSD calculated (cycle=3, 211 Cα atoms).

a

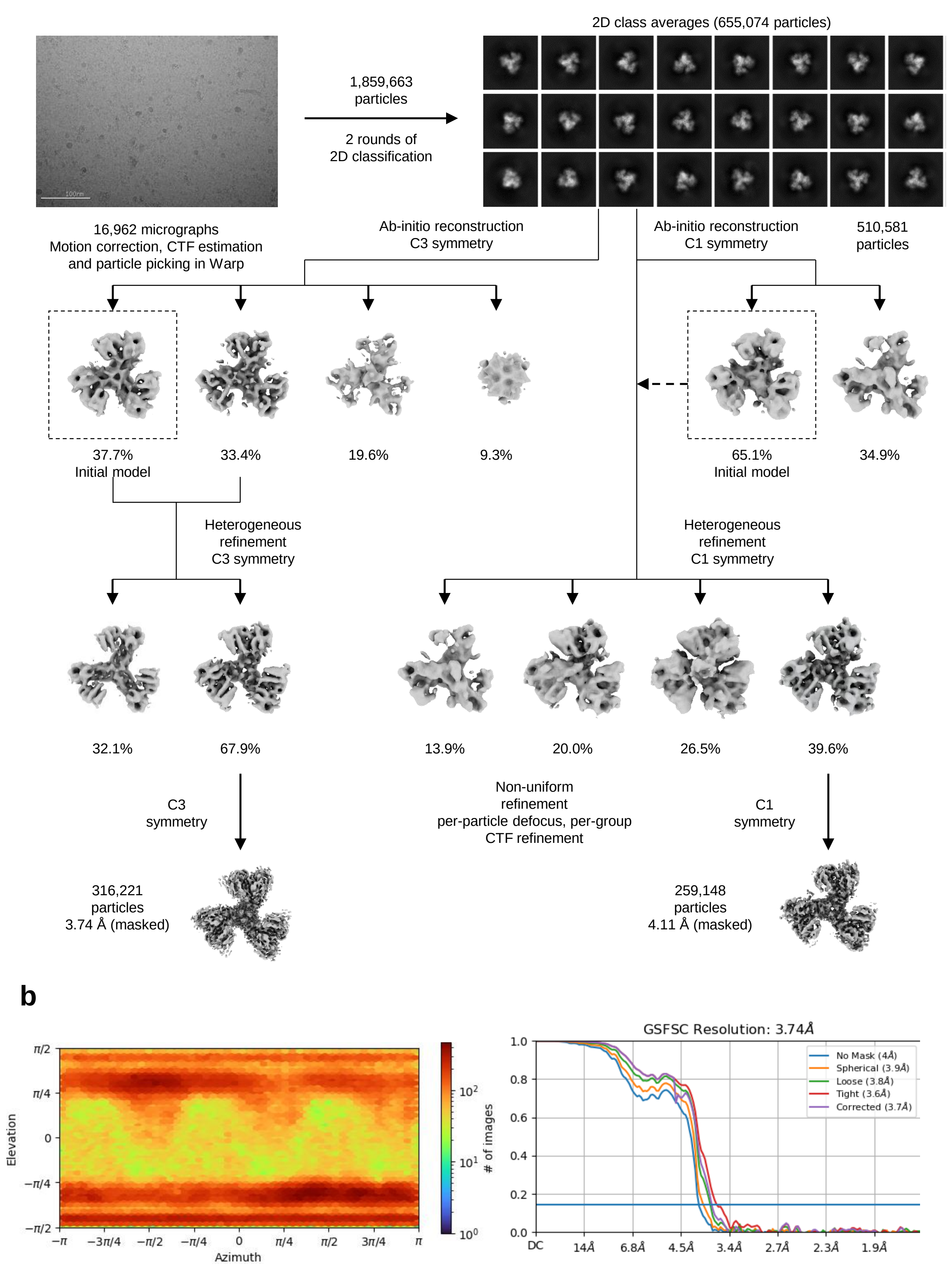
2D class averages (655,074 particles)
1,859,663
particles
2 rounds of
2D classification
16,962 micrographs
Motion correction, CTF estimation
and particle picking in Warp
Ab-initio reconstruction
C3 symmetry
Ab-initio reconstruction
C1 symmetry
510,581
particles
37.7%
Initial model
33.4%
19.6%
9.3%
65.1%
Initial model
34.9%
Heterogeneous
refinement
C3 symmetry
Heterogeneous
refinement
C1 symmetry
32.1%
67.9%
13.9%
20.0%
26.5%
39.6%
C3
symmetry
Non-uniform
refinement
per-particle defocus, per-group
CTF refinement
C1
symmetry
316,221
particles
3.74 Å (masked)
259,148
particles
4.11 Å (masked)
b
Elevation
Azimuth
# of images
GSFSC Resolution: 3.74Å
No Mask (4Å)
Spherical (3.9Å)
Loose (3.8Å)
Tight (3.6Å)
Corrected (3.7Å)
DC
14Å
6.8Å
4.5Å
3.4Å
2.7Å
2.3Å
1.9Å

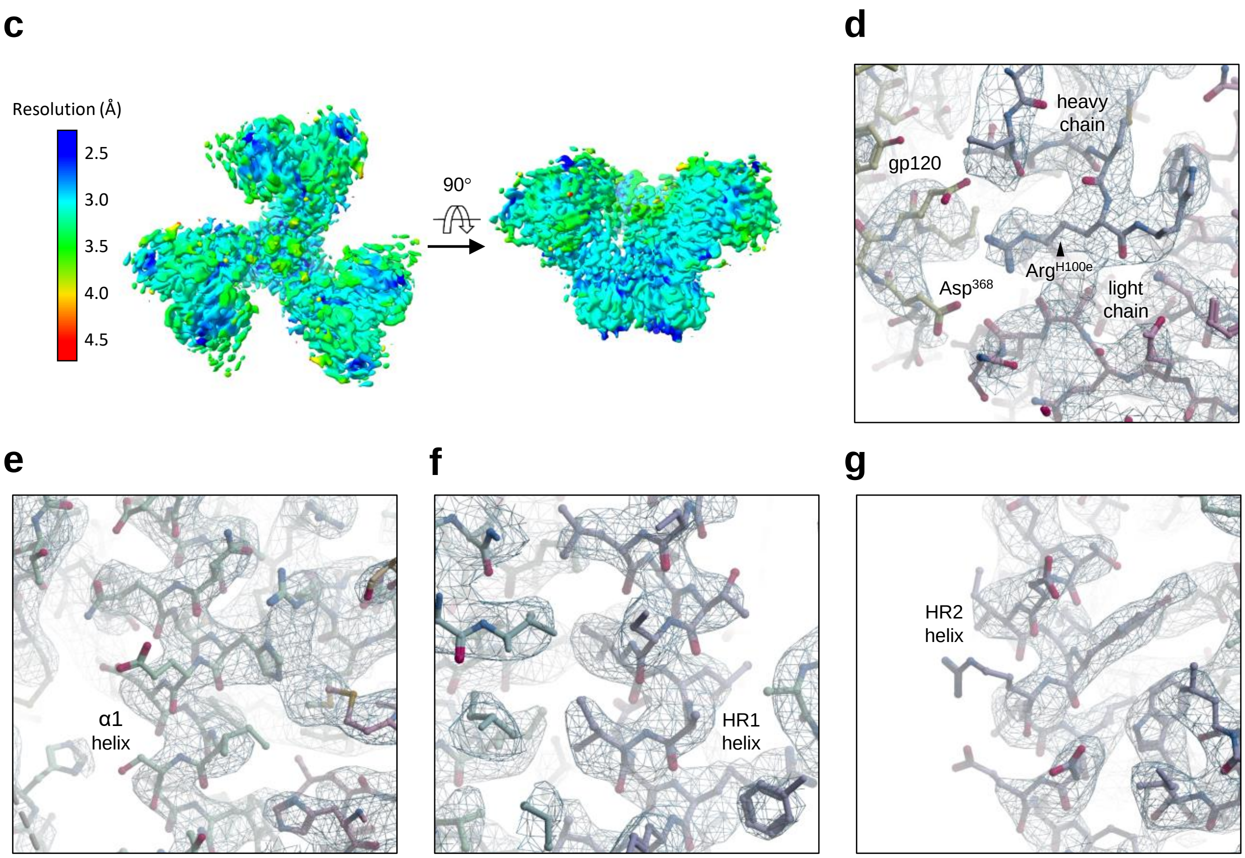

Supplementary Figure 5 | Cryo-EM details of the HmAb64 in complex with CNE40 SOSIP trimer. a Cryo-EM data processing workflow. **b** Angular distribution of the cryo-EM particles used in the reconstruction and resolution estimation generated with cryoSPARC using an FSC cutoff of 0.143. **c** Local resolution was estimated with cryoSPARC using the non-uniform refinement half maps. **d-g** Representative densities were shown for the interface between gp120 and HmAb64 (**d**), the α1 helix of gp120 (**e**), and a partial region of HR1 and HR2 of gp41 (**f** and **g**, respectively). The key residue in the CDR H3 loop of HmAb64, Arg^{H100e}, was indicated by an arrowhead in **d**.

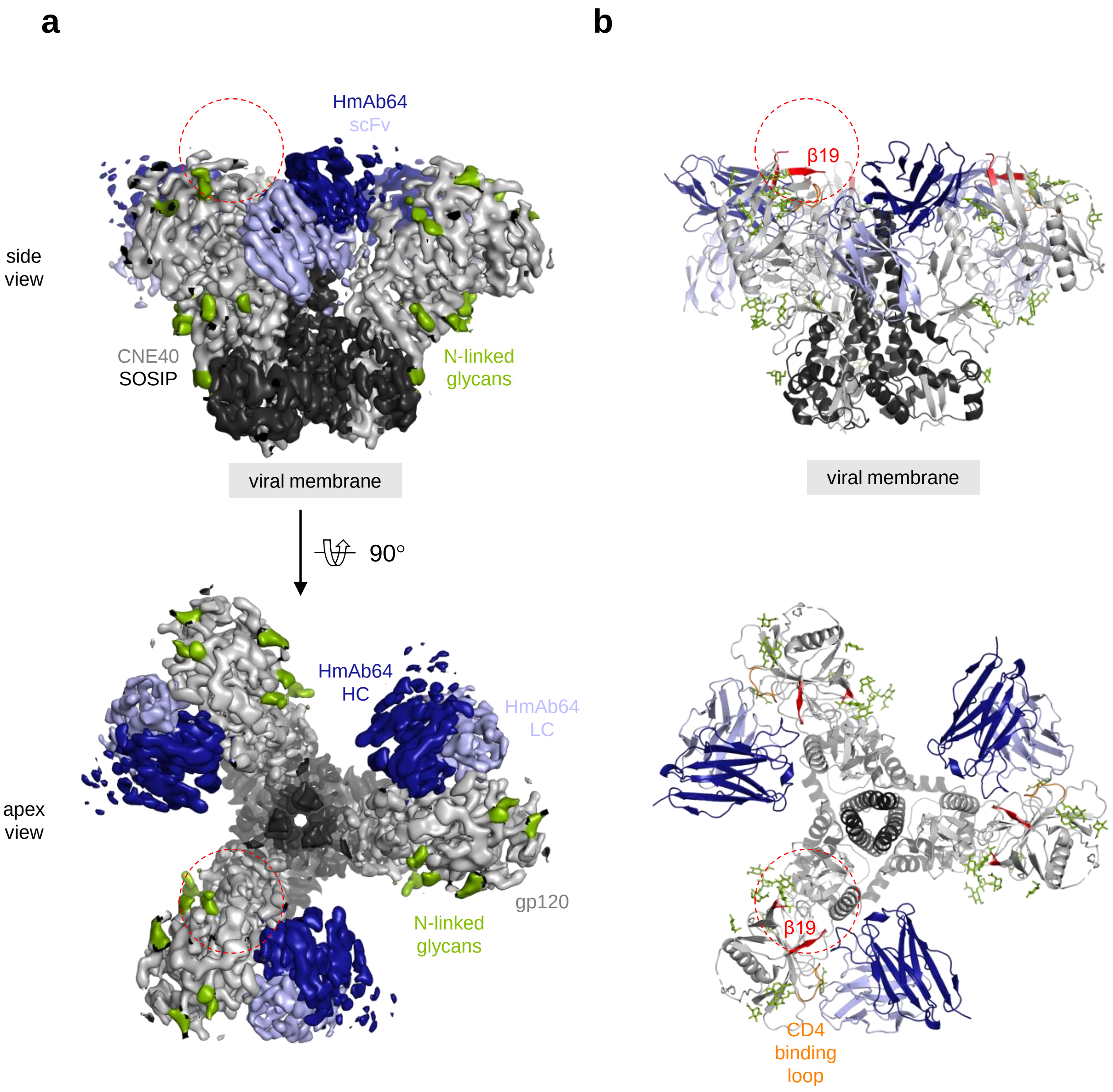

Supplementary Figure 6. Cryo-EM complex structure of HmAb64 scFv with a CNE40 SOSIP trimer. a-b Cryo-EM density (**a**) and ribbon (**b**) representations of the complex structure of HmAb64 with a SOSIP trimer. The regions corresponding to the gp120, gp41, and N-linked glycans were colored gray, dark gray and green, respectively, while the light chain (LC) and heavy chain (HC) of HmAb64 were colored dark and light blue, respectively. In **b**, each CD4-binding loop in the gp120 protomer was highlighted in orange. Due to conformational flexibility, the densities at the regions corresponding to the C4 region (within a dashed circle, including the β20/β21 loop) and the entire apical regions, including V1V2 and V3, were averaged out, likely caused by the binding of HmAb64.

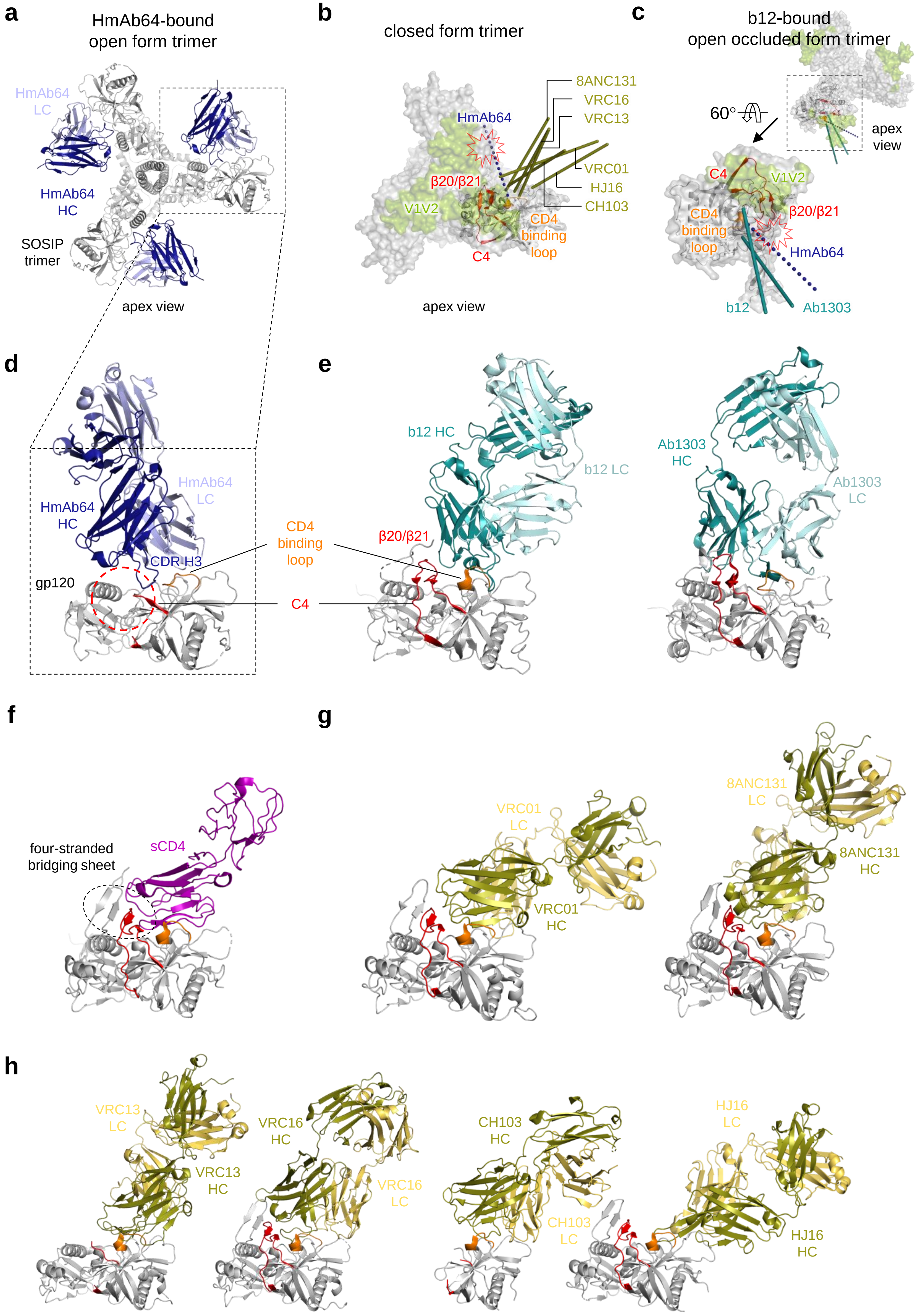
a
HmAb64-bound open form trimer
HmAb64 LC
HmAb64 HC
SOSIP trimer
apex view
b
closed form trimer
8ANC131
VRC16
VRC13
HmAb64
VRC01
HJ16
CH103
β20/β21
V1V2
CD4 binding loop
C4
apex view
c
b12-bound open occluded form trimer
60°
apex view
C4
V1V2
CD4 binding loop
β20/β21
HmAb64
b12
Ab1303
d
HmAb64 LC
HmAb64 HC
CDR H3
gp120
CD4 binding loop
C4
e
b12 HC
b12 LC
β20/β21
Ab1303 HC
Ab1303 LC
f
four-stranded bridging sheet
sCD4
g
VRC01 LC
VRC01 HC
8ANC131 LC
8ANC131 HC
h
VRC13 LC
VRC13 HC
VRC16 HC
VRC16 LC
CH103 HC
CH103 LC
HJ16 LC
HJ16 HC

Supplementary Figure 7 | A different angle of approach used by HmAb64 from other CD4bs nAbs. a Complex structure of HmAb64 with a SOSIP trimer revealed HmAb64 recognizes an open form Env trimer. **b-c** To illustrate the difference in the angle of approach to gp120 between HmAb64 and other CD4bs nAbs, HmAb64 was aligned (displayed in a dashed line to represent the direction of approach) onto both a closed form trimer (**b**, PDB 5FYK) and an occluded open form trimer (**c**, PDB 5VN8) with their respective representative CD4bs nAbs (in solid lines) while HmAb64 is incompatible with these trimer conformations due to an increased steric hindrance (red explosion lines). Each primary gp120 in **b** and **c** was shown in ribbon with C4 (including β20/β21, in red), CD4-binding loop (in orange) highlighted, and apical regions (V1V2 and V3) omitted for clarity, while the surface regions corresponding to V1V2 were colored in green. The direction of the antibody approach was calculated based on two reference points: the center of the coordinates of the Ca atoms of two conserved cysteine residues, Cys^{H92} and Cys^{L88}, from the heavy and light chain, respectively, and that of another two, Cys^{H140} and Cys^{L134}. **d-h** Structural models of HmAb64, CD4 and other CD4bs nAbs respectively bound to gp120 were used for comparison of the antibody approach angle in **b** and **c**, including: **d**) HmAb64 (this study, with the crystal structure of the HmAb64 Fab region (PDB 6W73) superimposed), **e**) open occluded form recognizing CD4bs nAbs, b12 (PDBs 5VN8 and 2NY7) and Ab1303 (PDBs 7TFN and 7RYU), with the apical regions (V1V2 and V3) removed for clarity, **f**) CD4 (PDB 8FYJ), which induces the Env trimer opening, featured by a unique structural element: the four-stranded bridging sheet (in dashed oval), **g**) representative VH gene-restricted CD4bs nAbs, VRC01 (PDB 3NGB) and 8ANC131 (PDB 4RWY), and **h**) representative CDR H3-dominated CD4bs nAbs, VRC13 (PDB 4YDJ), VRC16 (PDB 4YDK), CH103 (PDB 4JAN) and HJ16 (PDB 4YE4). All gp120 structures were aligned in the same orientation as the gp120 protomer boxed in **a**. Note that due to its distinct angle of approach to gp120, differing from other CD4bs nAbs and CD4, the recognition by HmAb64 results in a structural clash with the β20/β21 loop (indicated by the red dashed circle in **d**). This clash leads HmAb64 to bind to an open conformation of the Env trimer, which is different from the CD4-induced or open occluded form trimer.

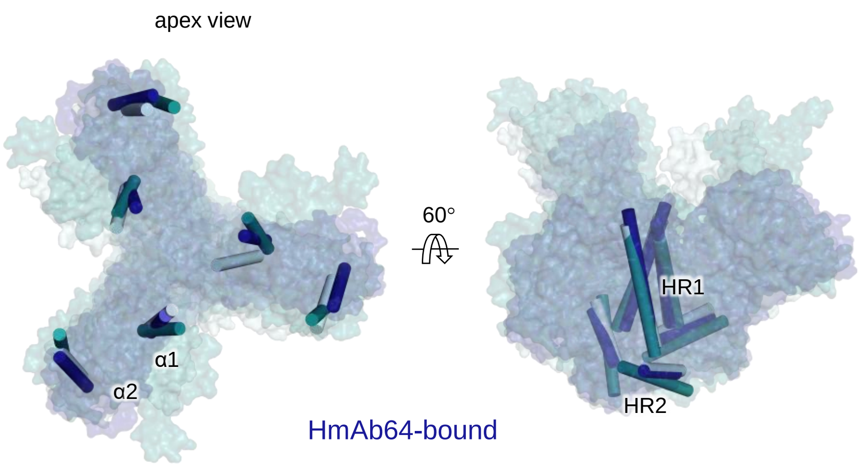

	vs b12-bound	vs Ab1303-bound
gp120	Outward rotation: 27º<br>Maximum displacement: 6 Å<br>RMS displacement: 1.8 Å	Outward rotation: 7-16º<br>Maximum displacement: 10 Å<br>RMS displacement: 2.4 Å
gp41	Maximum displacement: 14 Å<br>RMS displacement: 1.8 Å	Maximum displacement: 6 Å<br>RMS displacement: 2.0 Å

Supplementary Figure 8 | Comparison of the HmAb64-bound open form trimer with other open occluded form conformations. Structural alignment on the HR1 helices of the HmAb64-bound trimer (blue, this study) and open occluded form conformations recognized by b12 (teal, PDB 5VN8) and Ab1303 (light teal, PDB 7TFN). Each gp120 and gp41 subunit was represented by two conserved helices, α1/α2 and HR1/HR2, respectively, in cylinders.

Supplementary Table 1 | Data collection and refinement statistics.*

Data collection	Fab HmAb64 (PDB ID 6W73)
Space group	C121
Unit cell parameters	
a, b, c (Å)	91.79, 59.35, 75.57
a, b, g (°)	90, 96.22, 90
Resolution (Å)	2.80-45.63 (2.80-2.97)*
R_{merge}	8.5 (53.0)
I/sI	11.6 (2.84)
Completeness (%)	99.8
Redundancy	4.2
Refinement	
Resolution (Å)	2.80-45.63
Number of reflections	19,529
R_{work}/R_{free}	17.82/26.27
Number of atoms:	
Protein	3,308
Solvent	34
B-factors	
Protein (Å^2)	45.66

*Values in parentheses refer to the outer resolution shell.

Supplementary Table 2 | Cryo-EM Data Collection, Refinement and Validation Statistics for HmAb64 scFv in complex with HIV-1 CNE40 SOSIP trimer

	HmAb64 scFv in complex with CNE40 SOSIP (PDB 8TR3, EMD-41569)
Data collection and processing	
Magnification	105,000
Voltage (kV)	300
Electron exposure (e⁻/Å²)	56.76
Defocus range (μm)	-0.5 – -2.5
Pixel size (Å)	0.852
Symmetry imposed	C3
Final particle images (no.)	316,221
Map resolution (Å)	3.74
FSC threshold	0.143
Refinement	
Initial model used (PDB code)	6W73 (this study), 6CK9
Model resolution (Å)	3.74
FSC threshold	0.143
Map sharpening B factor (Å²)	-184.1
Model composition	
Non-hydrogen atoms	16401
Protein residues	2028
Ligands	NAG: 39
B-factors (Å², mean)	
Protein	81.06
Ligand	87.44
R.m.s. deviations	
Bond lengths (Å)	0.004
Bond angles (°)	0.676
Validation	
MolProbity score	2.08
Clash score	11.43
Rotamers outliers (%)	0.17
Ramachandran plot (%)	
Favored	91.46
Allowed	8.54
Outliers	0.00

Supplementary Table 3 | Primers used for RT-PCR and PCR

Primer name	Sequence
VH1 Leader -A	ATGGACTGGACCTGGAGGAT
VH1 Leader -B	ATGGACTGGACCTGGAGCAT
VH1 Leader-C	ATGGACTGGACCTGGAGAAT
VH1 Leader -D	GGTTCCTCTTTGTGGTGGC
VH1 Leader -E	ATGGACTGGACCTGGAGGGT
VH1 Leader -F	ATGGACTGGATTTGGAGGAT
VH1 Leader -G	AGGTTCCTCTTTGTGGTGGCAG
CH2-IgG universal	TCATTTACCCGGAGACAGGG
Vk1/2-Leader-A	ATGGACATGAGGGTCCCCGCTCAGC
Vk1/2-Leader-B	ATGGACATGAGGCTCCCGGCTCAGC
CL2-kappa	CTAACACTCTCCCCTGTTGAAGC
CH2-IgG-NheI	CTCTCCCTGTCTCCGGGTAAATGAGCTAGCCCGGGTGATAAGGA
VH1 LEADER-HindIII	GTCCAAGCTTGCCACCATGGACTGGACCTGGAGGAT
Vk1/2-Leader-HindIII	GTCCAAGCTTGCCACCATGGACATGAGGGTCCCCGCTCAGC
CL2-kappa-BamHI	GCGAGGATCCCTAACACTCTCCCCTGTTGAAGC
