# Supplementary material for: Human CD4-binding site antibody elicited by polyvalent DNA prime-protein boost vaccine neutralizes cross-clade tier-2-HIV strains
Source: Nat Commun. 2024 May 21;15:4301. doi: 10.1038/s41467-024-48514-8 (PMC11109196; doi:10.1038/s41467-024-48514-8)
Supplement: Supplementary file 4 — Source Data [file 41467_2024_48514_MOESM4_ESM.zip › Source Data_HmAb64 Supp Fig 2b and 2c.pdf]

# Supplementary figure 2 panels b and c source data

## Panel b titration and Panel c Leu3A inhibition

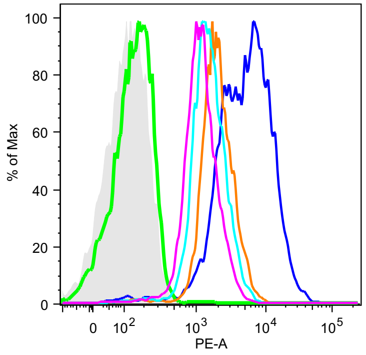


gp120 ADA
ADA+Leu3a
ADA+HmAb64 2ug
ADA+HmAb64 4ug
ADA+HmAb64 8ug

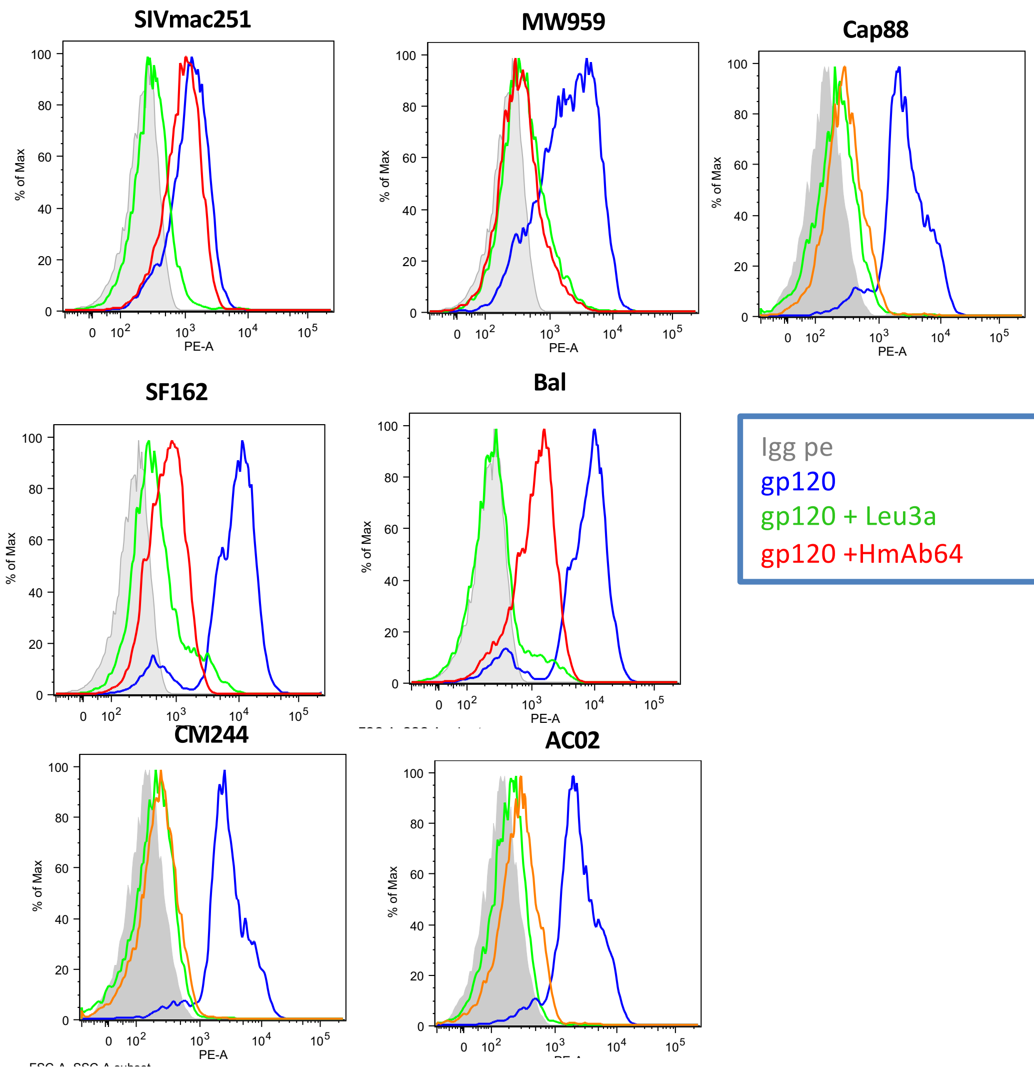


Igg pe
gp120
gp120 + Leu3a
gp120 +HmAb64
