# Supplementary figures and images for: Human CD4-binding site antibody elicited by polyvalent DNA prime-protein boost vaccine neutralizes cross-clade tier-2-HIV strains
Source: Nat Commun. 2024 May 21;15:4301. doi: 10.1038/s41467-024-48514-8 (PMC11109196; doi:10.1038/s41467-024-48514-8)

Figure 4B source data

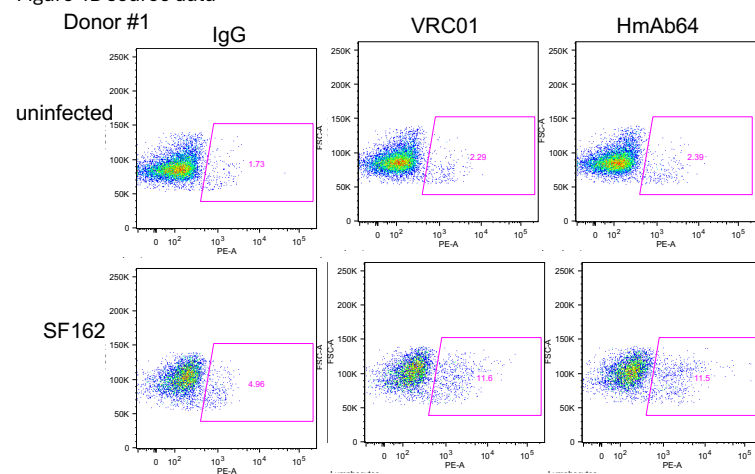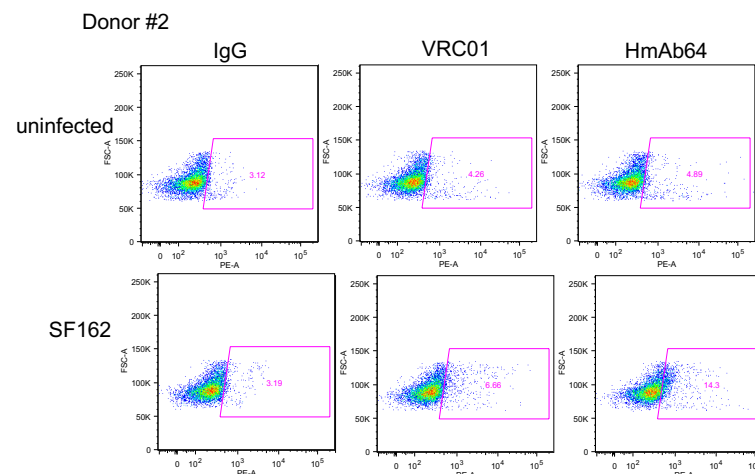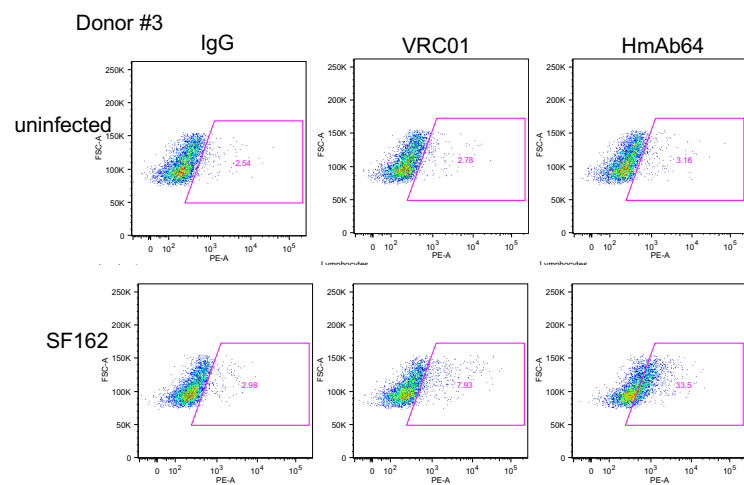

Supplement: Supplementary file 4 — Source Data [file 41467_2024_48514_MOESM4_ESM.zip › Source data_ HmAb64 Fig 4b.pdf]
